# Supplementary material for: Implantable silicon neural probes with nanophotonic phased arrays for single-lobe beam steering
Source: Commun Eng. 2024 Dec 18;3:182. doi: 10.1038/s44172-024-00328-8 (PMC11655650; doi:10.1038/s44172-024-00328-8)
Supplement: Supplementary file 2 — Supplementary information [file 44172_2024_328_MOESM2_ESM.pdf]

# Single-lobe beam steering in tissue with optical phased arrays on implantable neural probes: supplementary document

Fu Der Chen[1,2,3,*,†], Ankita Sharma[1,2,3,*,†], Tianyuan Xue[1,2], Youngho Jung[1], Alperen Govdeli[1,2], Jason C. C. Mak[1], Mandana Movahed[4], Homeira Moradi Chameh[4], Michael G. K. Brunk[1], Xianshu Luo[5], Hongyao Chua[5], Patrick Guo-Qiang Lo[5], Taufik A Valiante[2,3,4,6,7], Wesley D. Sacher[1,3], Joyce K. S. Poon[1,2,3,†]

[1]*Max Planck Institute of Microstructure Physics, Weinberg 2, 06120 Halle, Germany*

[2]*Department of Electrical and Computer Engineering, University of Toronto, 10 King's College Road, Toronto, Ontario M5S 3G4, Canada*

[3] *Max Planck-University of Toronto Centre for Neural Science and Technology*

[4] *Krembil Brain Institute, University Health Network, Toronto, Ontario, Canada*

[5]*Advanced Micro Foundry Pte Ltd, 11 Science Park Road, Singapore Science Park II, 117685, Singapore*

[6]*Division of Neurosurgery, Department of Surgery, Toronto Western Hospital, University of Toronto, Toronto, Ontario, Canada*

[7]*Institute of Biomedical Engineering, University of Toronto, Toronto, Ontario, Canada*

[*]These authors contributed equally to this work.

[†]Corresponding authors: fuder.chen@mail.utoronto.ca, ank.sharma@mail.utoronto.ca, joyce.poon@utoronto.ca

## Supplementary Note 1. Steering Arc Length Vs. Propagation Distance

In Supplementary Figure 1, we plot the sidelobe free steering arc length as a function of propagation distance. For distances $\leq$ 150 µm, the steering arc length of OPA I exceeds OPA II. However, OPA II achieves a greater steering range at larger propagation distances as expected from the relationships derived by the phase matching condition for a straight instead of concave grating. Across all propagation distances OPA III achieves the greatest sidelobe steering arc length.

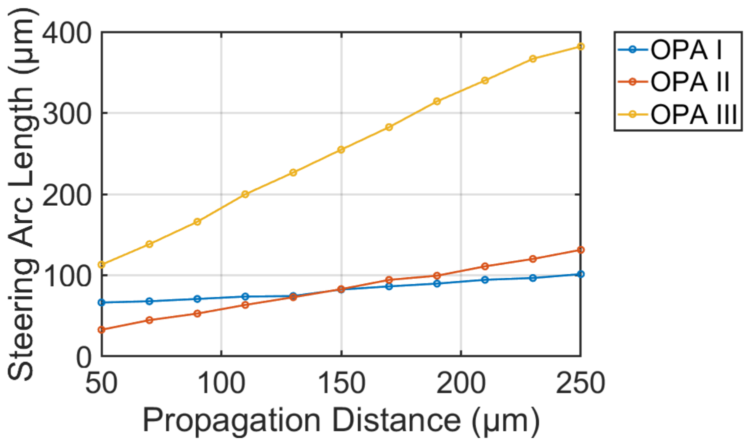


Supplementary Figure 1. Plot of steering arc length as a function of propagation distance (distance away from the OPA). We compare the steering arc length across the three blue OPA designs in the main manuscript (I, II, III) each with a different grating curvature.

## Supplementary Note 2. OPA Design Considerations And Scaling Limitations

In Supplementary Figure 2a, we annotate the key OPA parameters that along with the curvature of the slab grating, impact the device performance and footprint. The pitch ($d$) between waveguides in the end-fire array determines the in-plane steering range ($\pm max(\phi_{in})$) [1]. Additionally, the slab grating period ($\Lambda$) also contributes to the steering enhancement factor ($\Gamma$) given in Eqn. 2. The OPAs with the smallest footprints (OPAs Type II and IV) are constrained by the minimum bend radius and the need to maintain a reasonable pitch between the waveguide delay lines to minimize crosstalk. We select a bend radius that keeps the simulated insertion loss $<$ 3.5 dB/180° bend with an offset. We also choose the smallest possible pitch in the delay lines such that there is no more than -7 dB of coupling between two channels for a 100 µm propagation length. Simulations of both waveguide cross sections at a wavelength of $\lambda = 460$ nm are included in Supplementary Figure 2c and d for reference. In Supplementary Figure 2b, we demonstrate that given these constraints it is feasible to design OPA II to be more compact using thicker, higher index SiN waveguides. OPA II defined in 200 nm thick LPCVD instead of 150 nm thick PECVD SiN with a bend radius of 5 µm and delay line pitch of 0.7 µm would occupy a footprint of 0.0034 $mm^2$ rather than 0.007 $mm^2$. Consequently, eight of these devices could fit onto a 70 µm-wide shank as shown in Supplementary Figure 2e. Integrating additional

OPAs onto the shank would require a wider shank or smaller OPAs. Lastly, a final design consideration involves including the free propagation region (FPR) slab to enhance steering at propagation distances between 50-140 μm away from the OPA. Including an FPR into the design in Supplementary Figure 2b would increase the pitch between OPAs on the shank.

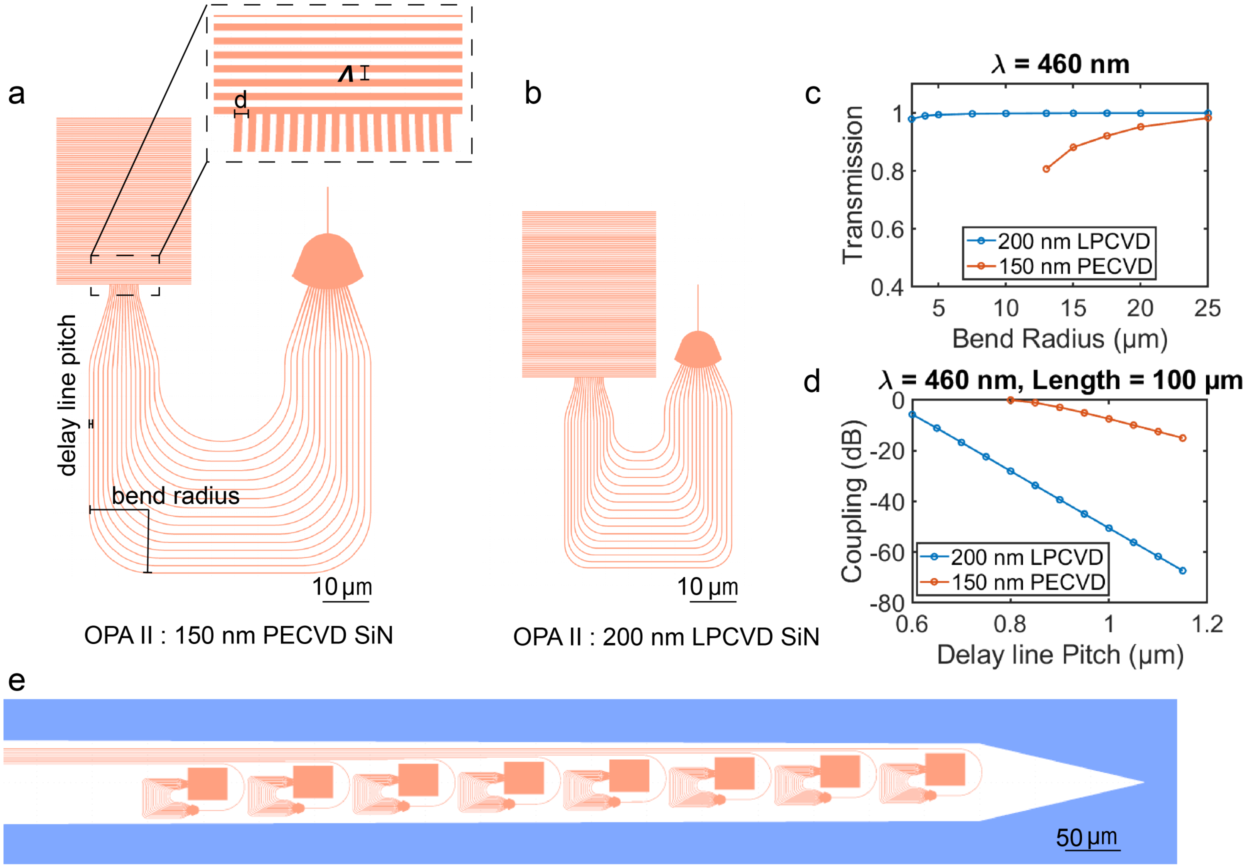


Supplementary Figure 2. **a** Schematic of the OPA Type II design defined in a 150 nm thick PECVD SiN layer. $d$ is the pitch between waveguide emitters in the end-fire phased array. $\Lambda$ is the period of the slab grating. Bend radius and delay line pitch are important parameters affecting the device footprint. **b** Schematic of OPA II defined in a 200 nm layer LPCVD SiN layer. The footprint is less than half the size of the design in **a**. **c** Simulated bend loss as a function of bend radius for the two waveguide cross sections. **d** Simulated crosstalk per 100 μm length as a function of delay line pitch for the two waveguide cross sections. **e** 70 μm wide shank design using the OPAs in **b**.

## Supplementary Note 3. Integration Density Comparison With Discrete Grating Emitters

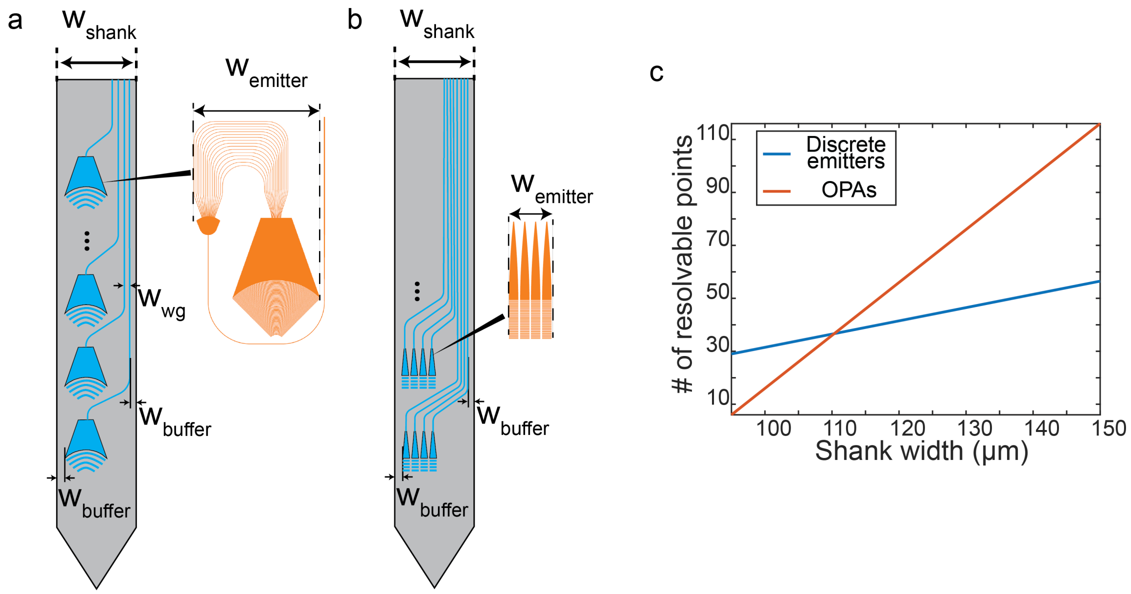


Supplementary Figure 3. Layout comparison between **a** the OPA probe with Type III emitters and **b** an equivalent neural probe featuring groups of 4 discrete grating emitters designed to replace a single OPA Type III emitter which achieved 4 resolvable points in fluorescein solutions. Each discrete grating emitter has a width of 6 μm and a gap of 1 μm between adjacent emitters. The critical widths that determine the number of resolvable points estimated with Eqn. S1 are labeled in the layout. **c** Comparison of the number of resolvable points achievable by the discrete grating emitter and the OPA designs under different shank widths.

This section analyzes the scaling benefits of the OPAs compared to discrete emitter designs. Supplementary Figure 3a and b show the layout comparison between a neural probe with OPA Type III emitters and a neural probe with groups of 4 discrete emitters in an array, offering the same number of resolvable points achieved by the OPA Type III emitter under non-scattering conditions at a propagation distance of 50 μm (4 resolvable points). As evident from the layout, the reduced routing space required for the OPA to generate multiple resolvable points offers a scaling benefit along the probe shank. The number of resolvable points achievable by both designs can be estimated with the equation below:

Supplementary Table 1. Parameters used for estimating the number of resolvable points achievable between the OPA and discrete grating emitter designs

| **Parameters** | **OPA design** | **Discrete emitter design** |
|---|---|---|
| $w_{emitter}$ (µm) | 82 | 27 |
| M | 4 | 1 |
| $w_{buffer}$ (µm) | 5 | |
| $w_{wg}$ (µm) | 2 | |

$$\# \text{ of addressable points} = M \times \frac{w_{\text{shank}} - w_{\text{emitter}} - 2 \times w_{\text{buffer}}}{w_{\text{wg}}} \tag{S1}$$

where $w_{shank}$ is the shank width, $w_{wg}$ is the waveguide routing pitch, $w_{buffer}$ is the buffer region of the components to the edge of the shank, $w_{emitter}$ is the width of the emitter and M denotes the number of resolvable points per emitter. These parameters are also annotated on Supplementary Figure 3a and b. In this comparison, we used a grating coupler design with a width of 6 µm and an array pitch of 7 µm. This pitch is comparable to the FWHM beam width of the grating emitter, as measured at a propagation distance of 50 µm in fluorescein [2], ensuring non-overlap beam profiles between neighboring emitters.

Supplementary Figure 3c compares the number of resolvable points achieved by the two designs as a function of shank width based on the parameters presented in Supplementary Table 1. The analysis reveals that the OPA design outperforms the discrete grating design in terms of resolvable points when the shank width exceeds 110 µm. It is important to note that this comparison provides a conservative estimate of the OPA design's scaling benefit, as the discrete grating design presented was optimized for a compact footprint with the same straight emission angles across all emitters. To match the area covered by the OPA steering range, discrete emitters can be tilted to enlarge their beam emission angles. However, this modification would increase the emitter width ($w_{emitters}$) of the discrete grating design, further increasing the scaling advantage of the OPA design.

## Supplementary Note 4. Scattering Simulation

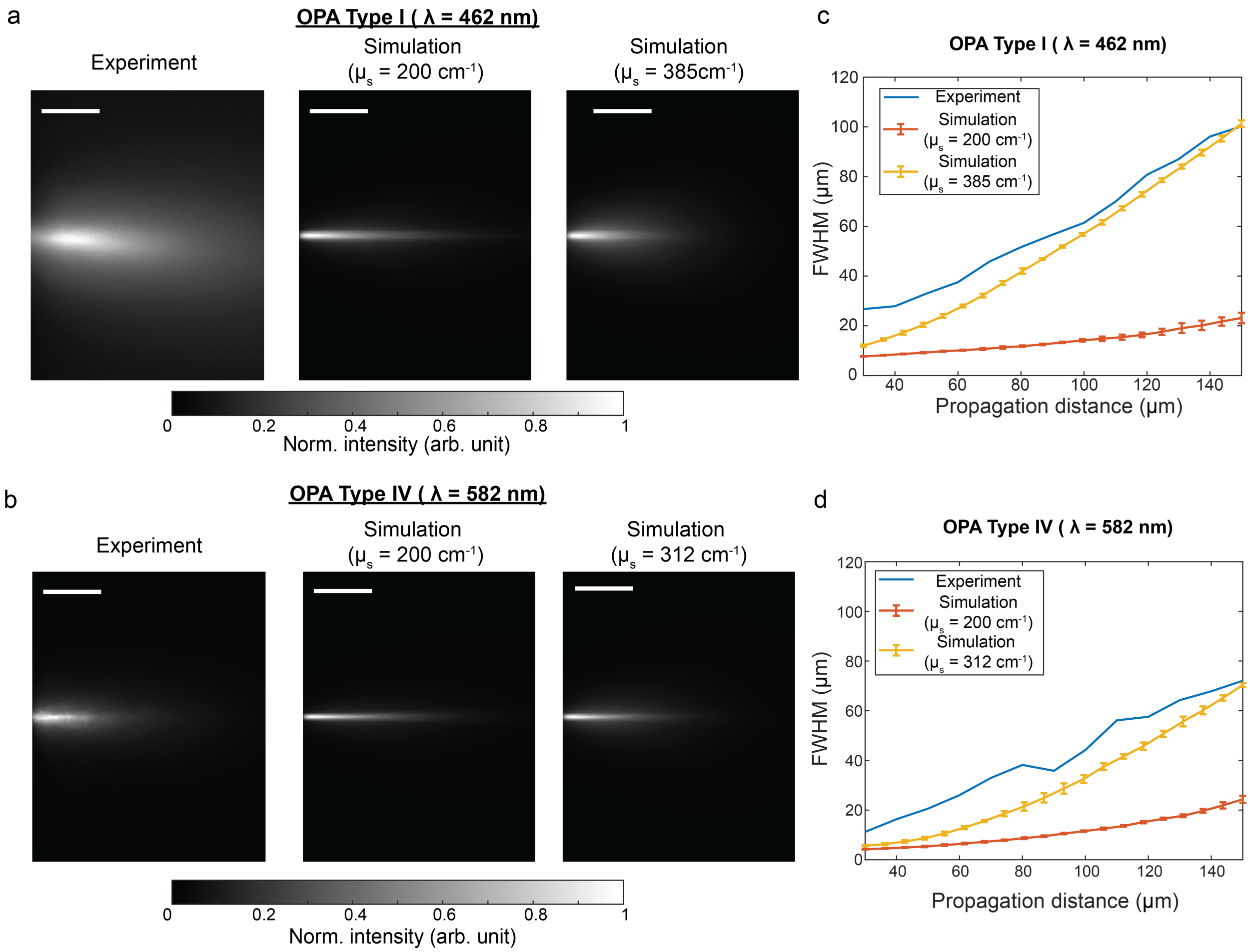


Supplementary Figure 4. Comparison of the simulated and experimental beam profiles for OPA Type I and IV in tissues. Experimental beam profile in fluorescent dye stained fixed mouse cortex slices and the simulated beam profiles at two different scattering coefficients for OPA **a** Type I and **b** Type IV. The scale bars are 50 μm. The FWHM beam width measured along the beam propagation distance of the beam profiles from **a** and **b** for OPA **c** Type I and **d** Type IV. The scattering simulations were repeated five times for each scattering setting, with each trial generated with new phase masks. The average beam widths (FWHM) were plotted, with error bars representing their standard deviations.

To simulate the beam profile in tissue, we employed the beam propagation method (BPM) described in [3]. This method models beam propagation using the angular spectrum method. A phase mask with small random phase variations is introduced after each propagation step

in the BPM to emulate the index variation across tissues, leading to beam scattering. The optical scattering properties of the medium can be adjusted by designing the phase masks following the design strategy detailed in [3]. The input fields of the OPAs to the BPM were extracted from the 3D FDTD simulation performed in Lumerical.

After obtaining the 3D beam intensity through the scattering simulation, we applied blurring filters to the transverse planes of the 3D beam, the planes parallel to the beam propagation direction, to emulate two beam broadening effects captured by the optical microscope: 1) the out-of-focus light away from the imaging focal plane and 2) the scattering of the fluorescent signal propagating through the top tissue layer covered on top of the OPA. We simulated this effect by convolving the transverse planes of the 3D beam intensity with a set of degraded point spread functions (PSF) obtained using a similar method described in [4]. The degraded PSF for each traverse plane was generated by forward propagating a diffraction-limited Gaussian point source [5](calculated with the 10× objective used in the experiment) with scattering to the tissue surface and then propagating in reverse direction without scattering to the predefined in-focus object plane. This reversed beam propagation approximates the point spread function detected by a microscope with a magnification of one. Any transverse planes located above the defined tissue surface were convolved with the PSF generated at the tissue surface assuming that light was incident on the surface. Subsequently, the processed transverse planes were summed together to form the simulated 2D images shown in Supplementary Figure 4a and b. To match the scattering properties of the mouse cortex, we set the scattering coefficient ($\mu_s$), absorption coefficient ($\mu_a$), and anisotropy factor (g) to 200 $cm^{-1}$, 0.62 $cm^{-1}$, and 0.89, respectively [6]. We also assumed that the in-focus object plane of the beam is positioned at 60 µm in depth within the tissue.

Supplementary Figure 4a and b compare the experimental and simulated beam profiles for OPA Type I and IV. However, significant discrepancies in FWHM beam width were observed between the simulated and the experimental results for the case with scattering properties that correspond to the mouse cortex (Supplementary Figure 4c and d). Better agreement was achieved by increasing the scattering coefficient to 385 $cm^{-1}$ and 312 $cm^{-1}$ for OPA Types I and IV. This result suggests that the optical scattering in the prepared fixed brain slices may be higher than the nominally reported value for fresh brain slices [6, 7], indicating that the beam width could be narrower when performing optogenetic stimulation in *in vivo* experiments.

## Supplementary References


[1] Y. Liu and H. Hu, Optica **9**, 903 (2022).

[2] F.-D. Chen, H. Wahn, T. Xue, Y. Jung, J. N. Straguzzi, S. S. Azadeh, A. Stalmashonak, H. Chua, X. Luo, P. Shah, H. M. Chameh, P. G.-Q. Lo, T. A. Valiante, W. D. Sacher, and J. K. S. Poon, in *Conference on Lasers and Electro-Optics* (Optica Publishing Group, 2022) p. JTh6A.7.

[3] Y. Li, J. Mertz, A. Devor, X. Cheng, L. Tian, S. Sakadžić, and D. A. Boas, Optics Letters **44**, 4989 (2019).

[4] T. Xue, A. Stalmashonak, F.-D. Chen, P. Ding, X. Luo, H. Chua, G.-Q. Lo, W. D. Sacher, and J. K. S. Poon, Scientific Reports **14**, 13812 (2024).

[5] B. E. A. Saleh and M. C. Teich, *Fundamentals of Photonics*, 3rd ed. (Wiley, 2019) p. 80.

[6] G. Yona, N. Meitav, I. Kahn, and S. Shoham, eNeuro **3**, 420 (2016).

[7] S. I. Al-Juboori, A. Dondzillo, E. A. Stubblefield, G. Felsen, T. C. Lei, and A. Klug, PLOS ONE **8**, e67626 (2013).
