# Supplementary material for: Implantable silicon neural probes with nanophotonic phased arrays for single-lobe beam steering
Source: Commun Eng. 2024 Dec 18;3:182. doi: 10.1038/s44172-024-00328-8 (PMC11655650; doi:10.1038/s44172-024-00328-8)
Supplement: Supplementary file 3 — Description of Additional Supplementary Files [file 44172_2024_328_MOESM3_ESM.pdf]

Description of Additional Supplementary Files

File name: Supplementary Movie 1

Description: OPA Type I beam steering in fluorescein solution (452 – 464 nm). The intensity of each frame is adjusted by a constant factor to improve visibility. The video is in real-time.

File name: Supplementary Movie 2

Description: OPA Type IV beam steering in brain tissue slice stained with Texas Red fluorescent dye (574 – 596 nm). The intensity of each frame is adjusted by a constant factor to improve visibility. The video is in real-time.
