# Supplementary material for: Implantable silicon neural probes with nanophotonic phased arrays for single-lobe beam steering
Source: Commun Eng. 2024 Dec 18;3:182. doi: 10.1038/s44172-024-00328-8 (PMC11655650; doi:10.1038/s44172-024-00328-8)
Supplement: Supplementary file 6 — Reporting Summary [file 44172_2024_328_MOESM6_ESM.pdf]

nature portfolio

Corresponding author(s): Fu-Der Chen,Ankita Sharma

Last updated by author(s): Oct 31, 2024

Reporting Summary

Nature Portfolio wishes to improve the reproducibility of the work that we publish. This form provides structure for consistency and transparency in reporting. For further information on Nature Portfolio policies, see our Editorial Policies and the Editorial Policy Checklist.

Statistics

For all statistical analyses, confirm that the following items are present in the figure legend, table legend, main text, or Methods section.

n/a	Confirmed	
☐	☒	The exact sample size (*n*) for each experimental group/condition, given as a discrete number and unit of measurement
☐	☒	A statement on whether measurements were taken from distinct samples or whether the same sample was measured repeatedly
☒	☐	The statistical test(s) used AND whether they are one- or two-sided<br>*Only common tests should be described solely by name; describe more complex techniques in the Methods section.*
☒	☐	A description of all covariates tested
☒	☐	A description of any assumptions or corrections, such as tests of normality and adjustment for multiple comparisons
☐	☒	A full description of the statistical parameters including central tendency (e.g. means) or other basic estimates (e.g. regression coefficient) AND variation (e.g. standard deviation) or associated estimates of uncertainty (e.g. confidence intervals)
☒	☐	For null hypothesis testing, the test statistic (e.g. *F*, *t*, *r*) with confidence intervals, effect sizes, degrees of freedom and *P* value noted<br>*Give P values as exact values whenever suitable.*
☒	☐	For Bayesian analysis, information on the choice of priors and Markov chain Monte Carlo settings
☒	☐	For hierarchical and complex designs, identification of the appropriate level for tests and full reporting of outcomes
☒	☐	Estimates of effect sizes (e.g. Cohen's *d*, Pearson's *r*), indicating how they were calculated

Our web collection on statistics for biologists contains articles on many of the points above.

Software and code

Policy information about availability of computer code

Data collection	Lumerical FDTD, Open Ephys GUI, IC Capture 2.5, FlyCapture2, MATLAB 2020b or2022a, Python, Arduino IDE
Data analysis	ImageJ (FIJI package), Python (3.9.16, 3.11.5, and 3.6.13), Spyking Circus, Phy, MATLAB 2020b or2022a.

For manuscripts utilizing custom algorithms or software that are central to the research but not yet described in published literature, software must be made available to editors and reviewers. We strongly encourage code deposition in a community repository (e.g. GitHub). See the Nature Portfolio guidelines for submitting code & software for further information.

Data

Policy information about availability of data

All manuscripts must include a data availability statement. This statement should provide the following information, where applicable:

- Accession codes, unique identifiers, or web links for publicly available datasets
- A description of any restrictions on data availability
- For clinical datasets or third party data, please ensure that the statement adheres to our policy

Raw electrophysiological data, beam profile images, and the source data for the figures are available at: https://doi.org/10.17617/3.1X2HCV.

Human research participants

Policy information about studies involving human research participants and Sex and Gender in Research.

Reporting on sex and gender	N/A
Population characteristics	N/A
Recruitment	N/A
Ethics oversight	N/A

Note that full information on the approval of the study protocol must also be provided in the manuscript.

Field-specific reporting

Please select the one below that is the best fit for your research. If you are not sure, read the appropriate sections before making your selection.

☒ Life sciences ☐ Behavioural & social sciences ☐ Ecological, evolutionary & environmental sciences

For a reference copy of the document with all sections, see nature.com/documents/nr-reporting-summary-flat.pdf

Life sciences study design

All studies must disclose on these points even when the disclosure is negative.

Sample size	The experiments conducted were not hypothesis-based, and therefore, we did not predetermine the sample size.
Data exclusions	The analysis did not exclude any relevant data.
Replication	OPA probe induced robust spiking activity with optogenetic stimulation over multiple trials and across 3 Thy1-ChR2-YFP mice. We presented one of the examples in Figure 5 of the main text.
Randomization	The experiments were not randomized.
Blinding	Experimenters were not blinded from the group.

Reporting for specific materials, systems and methods

We require information from authors about some types of materials, experimental systems and methods used in many studies. Here, indicate whether each material, system or method listed is relevant to your study. If you are not sure if a list item applies to your research, read the appropriate section before selecting a response.

Materials & experimental systems

n/a	Involved in the study	
☒	☐	Antibodies
☒	☐	Eukaryotic cell lines
☒	☐	Palaeontology and archaeology
☐	☒	Animals and other organisms
☒	☐	Clinical data
☒	☐	Dual use research of concern

Methods

n/a	Involved in the study	
☒	☐	ChIP-seq
☒	☐	Flow cytometry
☒	☐	MRI-based neuroimaging

Animals and other research organisms

Policy information about studies involving animals; ARRIVE guidelines recommended for reporting animal research, and Sex and Gender in Research

Laboratory animals	Adult male and female Thy1-ChR2-EYFP mice (The Jackson Laboratory, Bar Harbor, Maine, stock number 007612) were used in the optogenetic stimulation experiments and C57BL/6J mice were used for beam profile characterization in brain slices. All animals were kept in a vivarium maintained at 22 °C with a 12-h light on/off cycle. Food and water were available ad libitum.

Wild animals	No wild animals were involved in the experiment
Reporting on sex	This research does not involve gender study. Animal gender were randomly selected.
Field-collected samples	No samples were collected in the field in the experiment.
Ethics oversight	All procedures for in vivo experiments described in Methods were reviewed and approved by the animal care committees of the University Health Network in accordance with the Canadian Council on Animal Care guidelines. The surgical procedures to extract mouse brain samples for beam profile imaging were performed in accordance with German laws.

Note that full information on the approval of the study protocol must also be provided in the manuscript.
